# Border Screening for SARS

**DOI:** 10.3201/eid1101.040835

**Published:** 2005-01

**Authors:** Ronald K. St. John, Arlene King, Dick de Jong, Margaret Bodie-Collins, Susan G. Squires, Theresa WS Tam

**Affiliations:** * Public Health Agency, Ottawa, Ontario, Canada

**Keywords:** SARS, airport screening, thermal scanners, Health Alert Notices, quarantine, travelers, research

## Abstract

With the rapid international spread of severe acute respiratory syndrome (SARS) from March through May 2003, Canada introduced various measures to screen airplane passengers at selected airports for symptoms and signs of SARS. The World Health Organization requested that all affected areas screen departing passengers for SARS symptoms. In spite of intensive screening, no SARS cases were detected. SARS has an extremely low prevalence, and the positive predictive value of screening is essentially zero. Canadian screening results raise questions about the effectiveness of available screening measures for SARS at international borders.

## Abstract 2

Screening at national borders may not be effective in controlling SARS spread.

The first cases of severe acute respiratory syndrome (SARS) in Canada were recognized almost simultaneously in Vancouver and Toronto. In Toronto, the index case was diagnosed on March 13, 2003, when a cluster of SARS cases was identified and traced back to a traveler from Hong Kong, who arrived in Canada on February 23, 2003 (*1*). Two epidemic waves of SARS occurred in Toronto (*2*), which resulted in a national total of 251 probable cases with 43 deaths.

In the period that followed the initial reports of this new syndrome from Hong Kong and Vietnam, the disease spread rapidly to other countries by international airline travelers. On March 12, 2003, the World Health Organization (WHO) issued a global health alert (*3*) in response to the clusters of SARS in the Hong Kong Special Administrative Region, China, Vietnam (Hanoi City), and Singapore. WHO recommended increased national and international vigilance to recognize and report suspected cases of SARS. Subsequently, on March 15, 2003, WHO issued the first of several international travel advisories that identified major locations where SARS transmission was substantial and ongoing and advised international travelers about travel to affected areas. On March 27, 2003, WHO recommended that affected areas begin screening departing airline passengers for symptoms suggestive of SARS.

Health Canada monitored the spread of this new syndrome through the WHO-Health Canada Global Public Health Intelligence Network and regular communications with other international and Canadian provincial and territorial public health agencies. As soon as the rapid, international spread of SARS became evident and after SARS was imported into Canada, Health Canada undertook a variety of measures designed to limit importation and exportation of disease and the spread of the disease within Canada. We describe the measures taken to mitigate the spread of SARS and provide data on the effectiveness of these measures.

## Methods

Health Canada used a graduated, phased response to additional imported SARS cases. The response consisted of an information phase (March 18–May 14, 2003), a screening phase (May 14–July 5, 2003), and a special measures phase (March 13–July 5, 2003).

### Information Phase

To mitigate the risk of importing SARS cases from other internationally affected areas, Health Canada distributed passenger health alert notices (HANs) for incoming passengers from affected areas in Southeast Asia on March 18, 2003. On arrival, posters directed passengers to pick up health information about symptoms and signs of SARS and advised them to consult a physician if a SARS-like illness developed after their arrival in Canada. This information was printed in several languages on conspicuous, yellow, 8 ½” x 11” paper (referred to as “yellow cards”) and contained key telephone numbers.

The initial posters and yellow HANs were placed at arrival sites in the Vancouver International Airport and Toronto’s Pearson International Airport. They were quickly made available in 12 other airports that received international passengers who might have traveled from the Far East. HANs were provided to inbound passengers at 18 land border crossings between the United States and Canada. No record was kept of how many passengers picked up HANs.

With the advent of SARS transmission in Toronto, Health Canada implemented similar HANs in a different color (cherry) to mitigate the risk of exporting SARS cases. The cherry-colored HANs were distributed to persons departing for international destinations from Toronto’s Pearson International Airport. Passengers with symptoms or signs of SARS were asked to self-defer their travel. In these instances, Health Canada requested airlines to waive their policies on nonrefundable tickets, and while many did so, the refund and rescheduling policies and conditions were not uniform.

### Screening Phase

Because of the continuing outbreak in Toronto, domestic spread in other affected countries in Southeast Asia, and international spread to other countries, Health Canada intensified its initial response by instituting both inbound and outbound passenger screening to identify persons with symptoms or signs compatible with SARS. All passengers were now required to obtain, read, and respond to questions on yellow or cherry HANs. Three questions were added to both HANs: Do you have a fever? Do you have one or more of the following symptoms: cough, shortness of breath, difficulty breathing? Have you been in contact with a SARS-affected person in the last 10 days? All passengers were required to circle “yes” or “no” responses. Their responses were verified either by customs officials (for inbound passengers) or by airline check-in agents (for departing passengers from Toronto Pearson Airport). Quality control checks (random sampling and spot checks of prescribed procedures) were instituted to ensure compliance by those responsible for verifying passenger responses. For example, during a 1-week period, 82% of departing passengers received a cherry card at check in, and 73% were questioned about their responses by the check-in ticket agent.

Secondary screening procedures were established for all passengers who answered yes to any of the questions. It was mandatory for any such passenger to be referred to a screening nurse who administered a standard in-depth questionnaire and protocol. The secondary screening protocol included reasons for assessment, symptoms present at time of assessment, oral temperature, and defined criteria for disposition. On the basis of the responses elicited in the protocol, a passenger was released or referred to a predetermined hospital for an in-depth medical evaluation.

In parallel to these measures, Health Canada initiated a pilot study on May 8, 2003, on the use of infrared thermal scanning machines to detect temperatures >38°C in selected international arriving and departing passengers at Vancouver’s International and Toronto’s Pearson International airports. Thermal scanning complemented other measures in the overall screening process by helping to triage the large volume of passengers who transit airports. Any passenger with an elevated temperature reading was referred to the screening nurse for confirmation, completion of the screening protocol, and referral to hospital, if necessary.

### Special Measures

#### Passenger Contact Tracing

With previous documentation of transmission of tuberculosis on long flights (*4*,*5*), Health Canada initiated passenger contact tracing to identify any secondary transmission associated with air travel. Health Canada’s protocols for airplane passenger contact tracing evolved throughout the SARS outbreak and were updated as new information became available. From March 13 to March 21, 2003, contact tracing of passengers included follow-up of passengers seated in the same row, 2 rows in front, and 2 rows behind someone with a probable case who was symptomatic while in flight. As of March 22, airplane passenger contact tracing was expanded to include persons with suspected cases who were symptomatic while in flight. As of March 31, contact tracing was expanded again to include all passengers on a given flight with a probable or suspected case who were symptomatic while in flight (*6*).

Because of the lack of internationally accepted standards for developing and retaining passenger manifests, Health Canada personnel encountered excessive delays in obtaining the manifests from various airlines. In response, Health Canada initiated a traveler contact information form that collected location information and that all inbound passengers were required to complete before arrival. Upon landing, all forms were collected from passengers by Health Canada personnel and retained for possible contact tracing if a case was subsequently identified. The traveler contact information rorm reduced the time for securing the manifest from weeks to 2 days.

All screening measures (HANs, thermal screening, and traveler contact information form) continued after July 5, 2003, when WHO declared that SARS outbreaks had been contained worldwide. This report only includes data up to that date, when international movement of SARS was a real possibility.

## Results

No attempt was made to evaluate the initial information phase. Data were collected for the screening phase. Table 1 summarizes the screening results for inbound and outbound HAN screening measures. As of July 5, 2003, a total of 1,172,986 persons received either yellow or cherry HANs. A total of 2,889 persons answered yes to at least 1 screening question on the HAN and were referred to secondary screening according to protocol. None of the 411 outbound passengers who were referred for secondary screening in Toronto were asked to defer their travel.

**Table 1:** Air passenger screening results for HAN* given to passengers on outbound and inbound flights, May 14–July 5, 2003

Measure	Location	Cumulative results			Comment
		Persons given HAN (n)	Persons referred (n)	Final disposition	
HAN outbound	Toronto	495,492	411	All cleared	All international flights departing from Toronto required to use cherry-colored HAN
HAN inbound	Toronto	349,754	1,264	All cleared	All international flights arriving in Toronto (70+ airlines) and Vancouver (100+ airlines) required to use yellow-colored HAN
	Vancouver	115,227	669	All cleared	
	Other	212,513	545	All cleared	
Total		1,172,986	2,889	All cleared	

*HAN, health alert notice.

All persons were cleared, and none were referred for additional medical examination. In addition, 763,082 persons (467,870 inbound and 295,212 outbound) were screened by the thermal scanners (Table 2). Only 191 persons had an initial temperature reading >38°C and were referred for secondary evaluation. No data were collected systematically to correlate thermal scanner results with results of temperature taking by secondary screening nurses. Some of the persons arriving or departing Toronto and Vancouver airports were screened by both HAN and thermal scanning measures.

**Table 2:** Air passenger screening results for thermal imaging scanners, May 16–July 5, 2003

Location	Measure	Cumulative results		
		Persons scanned (n)	Persons referred (n)	Final disposition
Toronto	Inbound	355,532	83	All cleared
	Outbound	281,959	94	All cleared
	Subtotal	637,491	177	All cleared
Vancouver	Inbound	112,338	12	All cleared
	Outbound	13,253	2	All cleared
	Subtotal	125,591	14	All cleared
Total		763,082	191	All cleared

During this period, no screening measure put in place by Health Canada detected any cases of SARS at border entry points. Careful analysis of the travel histories of suspected and probable SARS patients who traveled to Canada showed that persons became ill after arrival and would not have been detected by airport screening measures.

Table 3 summarizes the travel histories of persons departing Canada and subsequently diagnosed with a SARS-like illness. Health Canada collaborated with many international public health authorities to document travel and illness histories of possible SARS patients who departed Canada and were diagnosed and reported internationally (*7*–*9*). Health Canada investigated >40 such reports, of which 11 are now attributed to Canada (*10*). In all but 2 cases (cases 2 and 11), onset of illness occurred after departure from Canada. Of these 11 persons who traveled from Canada, all met the WHO probable SARS case definition. Only 3 of these case-patients met the Canadian probable case definition. Another 3 case-patients would meet the Canadian geo-linked case definition; 1 case met the Canadian “person under investigation” category; and 4 case-patients did not meet any Canadian SARS case definition. Of the 3 case-patients who did meet the Canadian definition, none would have been detected by exit screening. Only 2 (patients 2 and 11) of the 11 persons had symptoms at the time of travel, but both would have been cleared by the criteria established in the secondary screening protocol.

**Table 3:** Travel histories of persons departing from Canada in whom a SARS-like illness was subsequently diagnosed*

Case no.	Age	Sex	Depart date, Toronto	Onset of illness	Probable SARS†	Link‡	Remarks
1	3	F	March 28	March 31	Yes	No	PCR negative for SARS-CoV
2	5	M	March 28	March 25	Yes	No	No evidence of pneumonia, PCR negative for SARS-CoV
3	1	M	March 28	April 1	Yes	No	No evidence of pneumonia, PCR negative for SARS-CoV
4	26	F	March 31	April 3	Yes	No	PCR and serologic test results negative for SAR-CoV
5	52	M	April 1	April 3	Yes	Yes	Traveled by car, PCR and serologic test results positive for SARS-CoV
6	46	F	April 3	April 6	Yes	Yes	Fatal SARS case
7	24	M	April 28	April 30	Yes	No	Serologic and PCR test results negative for SARS-CoV
8	28	F	April 24	May 3	Yes	No	No laboratory results available
9	29	M	May 10	May 13	Yes	No	Traveled by car, PCR negative and acute serologic test results negative for SARS-CoV (convalescent-phase serologic test results not available)
10	47	M	May 17	May 24	Yes	Yes	Acute–phase serologic test results positive for SARS-CoV
11	25	M	July 14	July 7	Yes	No	Laboratory results unavailable

*SARS, severe acute respiratory syndrome; F, female; M. male; SARS-CoV, SARS-associated coronavirus; PCR, polymerase chain reaction. †Met the World Health Organization probable SARS case definition at time of illness. ‡Is an epidemiologic link to another SARS case established?

We identified 18 symptomatic probable or suspected SARS patients on 29 flights (10 patients traveled on >2 flights). No documented transmission was identified. Detailed results of Canada’s airplane passenger contact tracing can be found elsewhere (*6*).

## Discussion

Patterns of international travel continue to increase in complexity and volume. In Canada, >18 million persons enter annually by air; 91% arrive at 6 international airports. Similarly, a large number depart from several international airports. Additionally, because of an open land border with the United States, ≈100 million persons cross the land border in both directions annually.

With travel to Canada from anywhere in the world taking <24 hours, the possibility of detecting a dangerous infectious disease at border points of entry is challenging. Given the relatively short travel time, detecting persons at the border who are incubating any of the known infectious disease pathogens is unlikely. The absence of symptoms or signs of infection and a corresponding lack of specific, extremely rapid, easy-to-use diagnostic tests make border detection of infectious diseases unlikely.

The effectiveness of screening measures for detecting SARS cases at border points of entry was limited by 2 factors. First, screening measures themselves, i.e., HAN questionnaires and thermal scanning machines, were nonspecific for SARS. Second, the prevalence of SARS among international passengers arriving or departing from Canada was low. For example, 5 SARS patients entered Canada from March through May. None of these patients had signs or symptoms during transit through airports. If the same rate of entry were to continue for 1 year, then 20 cases might be expected among the 18 million persons entering the country annually, for a prevalence of ≈1.1 SARS cases per 1 million passengers. For such a rare disease, the positive predictive value of a positive screening result is essentially zero. The results demonstrate that available screening measures are not effective for detecting SARS. Despite extending screening measures to all arriving air passengers, no SARS cases were identified. These findings raise questions about whether such measures are effective tools for detecting and controlling the spread of SARS, and whether, from a public health point of view, other, more effective, strategies might exist.

Instituting infectious disease screening procedures at border points of entry could have advantages. For example, easily visible measures, such as thermal scanning machines, may generate a sense of confidence or reassurance that disease will be detected and prevented from entering the country. No data are available to assess whether or not the measures implemented at the airports actually generated confidence or reassurance in the public. Given the poor positive predictive value of available SARS screening measures, any sense of reassurance might be quickly dispelled when the first case is detected in spite of screening measures.

We conclude that available screening measures for SARS were limited in their effectiveness in detecting SARS among inbound or outbound passengers from SARS-affected areas. We suggest that in-country, acute-care facilities (hospitals, clinics, and physicians’ offices) are the de facto point of entry into the healthcare system for travelers with serious infectious diseases. If a visitor or returning citizen becomes ill after arriving in Canada, he or she will likely seek medical care in clinics or emergency rooms. Acute-care facilities must to consider travel histories of all patients with suspected infectious diseases and implement standard precautions and infection control measures.

An estimated Can$7.55 million was invested in airport screening measures from March 18 to July 5. Rather than investing in airport screening measures to detect rare infectious diseases, investments should be used to strengthen screening and infection control capacities at points of entry into the healthcare system. Additional useful measures could focus on public education about infectious disease prevention and care.
